# Investigation into the Physiological Significance of the Phytohormone Abscisic Acid in *Perkinsus marinus*, an Oyster Parasite Harboring a Nonphotosynthetic Plastid

**DOI:** 10.1111/jeu.12379

**Published:** 2016-11-28

**Authors:** Hirokazu Sakamoto, Shigeo Suzuki, Kisaburo Nagamune, Kiyoshi Kita, Motomichi Matsuzaki

**Affiliations:** ^1^ Department of Biomedical Chemistry, Graduate School of Medicine The University of Tokyo 7‐3‐1 Hongo, Bunkyo‐ku Tokyo 113‐0033 Japan; ^2^ Faculty of Life and Environmental Sciences University of Tsukuba 1‐1‐1 Tennodai, Tsukuba Ibaraki 305‐8577 Japan; ^3^ Department of Parasitology National Institute of Infectious Diseases 1‐23‐1 Toyama Shinjuku‐ku Tokyo 162‐8640 Japan; ^4^ School of Tropical Medicine and Global Health Nagasaki University 1‐12‐4 Sakamoto Nagasaki 852‐8523 Japan; ^5^ Present address: Health Research Institute National Institute of Advanced Industrial Science and Technology (AIST) 2217‐14 Hayashi‐cho Takamatsu Kagawa 761‐0395 Japan

**Keywords:** Apicomplexa, endosymbiosis, isopentenyl pyrophosphate, mass spectrometry, Perkinsozoa, secondary plastid

## Abstract

Some organisms have retained plastids even after they have lost the ability to photosynthesize. Several studies of nonphotosynthetic plastids in apicomplexan parasites have shown that the isopentenyl pyrophosphate biosynthesis pathway in the organelle is essential for their survival. A phytohormone, abscisic acid, one of several compounds biosynthesized from isopentenyl pyrophosphate, regulates the parasite cell cycle. Thus, it is possible that the phytohormone is universally crucial, even in nonphotosynthetic plastids. Here, we examined this possibility using the oyster parasite *Perkinsus marinus*, which is a plastid‐harboring cousin of apicomplexan parasites and has independently lost photosynthetic ability. Fluridone, an inhibitor of abscisic acid biosynthesis, blocked parasite growth and induced cell clustering. Nevertheless, abscisic acid and its intermediate carotenoids did not affect parasite growth or rescue the parasite from inhibition. Moreover, abscisic acid was not detected from the parasite using liquid chromatography mass spectrometry. Our findings show that abscisic acid does not play any significant roles in *P. marinus*.

PLASTIDS arose from primary endosymbiosis and have spread to distinct eukaryotic lineages by secondary and tertiary endosymbioses (Archibald 2009; Keeling 2010). This expansion by a series of endosymbioses suggests that photosynthesis is a “delicious” capability in the struggle for survival. However, some plastid‐harboring organisms have lost photosynthetic ability during their evolution, e.g. in holoparasitic plants (Krause 2008), in algae such as *Helicosporidium* (Tartar 2013) and *Euglena longa* (*Astasia longa*) (Hachtel 1996), and in apicomplexan parasites such as *Plasmodium* spp. and *Toxoplasma gondii* (Kohler et al. 1997; McFadden et al. 1996). Interestingly, most of these organisms still retain plastids, suggesting that the organelles may have other cryptic essential functions besides photosynthesis. It is therefore necessary to elucidate the bona fide function of these organelles to fully comprehend the retention of plastids.

Plastids in apicomplexan parasites, termed apicoplasts, are the best‐studied example of nonphotosynthetic plastids; these parasites cause a range of human and veterinary diseases, and the plastid may serve as a promising drug target (Fichera and Roos 1997; He et al. 2001; Sato and Wilson 2005). The genomes of *T. gondii* and *Plasmodium* spp. indicate that the fatty acid, heme, iron‐sulfur cluster, and isopentenyl pyrophosphate (IPP) biosynthesis pathways are conserved in their apicoplasts (Fleige et al. 2010). IPP biosynthesis is an essential function of the apicoplast in the blood stage of *Plasmodium falciparum* (Yeh and DeRisi 2011) and in the asexual stage of *T. gondii* (Nair et al. 2011). IPP is a versatile precursor of a large number of compounds (Gershenzon and Dudareva 2007), of which we have focused on the apocarotenoid phytohormone abscisic acid (ABA) because it has a fundamental role in plastid‐harboring organisms. In higher plants, ABA plays an important role in seed dormancy, embryo development, and adaptation to environmental stresses, most notably drought (Hauser et al. 2011). In addition to plants, algae, and cyanobacteria (Hartung 2010; Nambara and Marion‐Poll 2005), *T. gondii* also produces ABA and exploits this compound in cell cycle regulation (Nagamune et al. 2008); ABA is necessary for the parasite to egress from host cells, and inhibition of ABA production induces cyst formation in the asexual stage. We therefore hypothesized that ABA biosynthesis is one of the molecular bases for retention of plastids even when photosynthetic ability has been lost.

To investigate the physiological significance of ABA in organisms harboring nonphotosynthetic plastids, we focused on *Perkinsus marinus*, a protozoan parasite of the Eastern oyster (*Crassostrea virginica*) that retains a nonphotosynthetic plastid (Grauvogel et al. 2007; Joseph et al. 2010; Matsuzaki et al. 2008; Stelter et al. 2007). The parasite is a member of the Myzozoa, along with Dinoflagellata and Apicomplexa (Hoppenrath and Leander 2009), and it is inferred from molecular phylogeny that their common ancestor was photosynthetic, and that the photosynthetic ability was lost independently in *Perkinsus* and apicomplexans (Bachvaroff et al. 2014; Janouskovec et al. 2010; Moore et al. 2008). Thus, a comparison of plastid functions of *Perkinsus* spp. with those of Apicomplexa is expected to be an effective first step in identifying the universality/diversity of nonphotosynthetic plastid evolution. Importantly, the IPP biosynthetic pathway is the only completely conserved plastid pathway identified in the *P. marinus* genome to date, and a recent study has shown that an ABA biosynthetic inhibitor, fluridone, prevents growth of *P. marinus* (Shridhar et al. 2013), suggesting that the parasite may biosynthesize ABA from IPP (Fig. 1). However, there is no experimental evidence of ABA production or its physiological significance in *Perkinsus* spp. Therefore, in this study, we tried to determine the production and significance of ABA in *P. marinus*.

**Figure 1:**
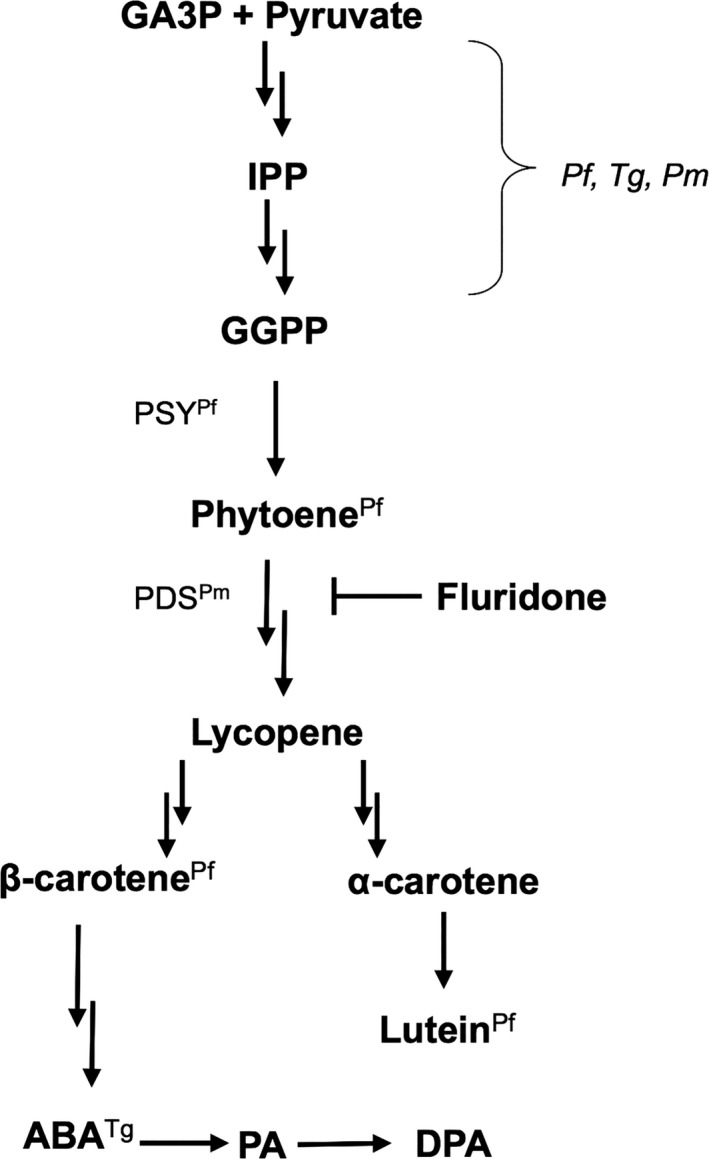
The schematic pathway of carotenoid‐ABA biosynthesis. Genes encoding the enzymes for GGPP biosynthesis from GA3P and pyruvate are conserved in *Plasmodium falciparum* (*Pf*), *Toxoplasma gondii* (*Tg*), and *Perkinsus marinus* (*Pm*). Downstream of GGPP, metabolites and enzymes identified in *Pf*,* Tg*, or *Pm* are indicated by the superscripts. Two candidate genes for PDS have been predicted in *Pm* (NCBI Gene IDs: 9037345 and 9041063). The double arrows indicate multiple enzymatic reactions. GA3P, glyceraldehyde 3‐phosphate; IPP, isopentenyl pyrophosphate; GGPP, geranylgeranyl pyrophosphate; PSY, phytoene synthase; PDS, phytoene desaturase; ABA, abscisic acid; PA, phaseic acid; DPA, dihydrophaseic acid.

## Materials and Methods

### Parasites and culture conditions

The *P. marinus* strain CRTW‐3HE was purchased from the American Type Culture Collection (ATCC 50439) and maintained at 26 °C in ATCC medium 1886. Lipid mixture (1,000×) (Sigma‐Aldrich, Saint Louis, MO) and Instant Ocean Sea Salt (Aquarium Systems, Sarrebourg, France) were used as substitutes for 100× lipid concentrate and Synthetic Seawater (Sigma‐Aldrich), respectively.

### Cell viability assay

The cell viability of *P. marinus* was assessed using the Cell Counting Kit‐8 (Dojindo Laboratories, Kumamoto, Japan) as previously reported (Sakamoto et al. 2016), and always three independent assays were performed in triplicate. For the growth inhibition assay, 100 mM fluridone (Fluka, Riedel‐de Haën, Germany) dissolved in dimethyl sulfoxide (DMSO) was added to each test well in the plate to yield the indicated final concentration. Absorbance of vehicle control at 0 and 3 d were defined as 0 and 100% of parasite growth, respectively. A dose–response curve was fitted for each assay using GraphPad Prism 6.0 (GraphPad Software Inc., San Diego, CA) with the built‐in model “log(inhibitor) vs. response” by constraining the Bottom plateau to 0. The mean IC_50_ with 95% confidence interval (CI) was then calculated from three independent assays.

For a rescue assay, β‐carotene (Wako Pure Chemical Industries, Osaka, Japan), lycopene (Wako Pure Chemical Industries), and ABA (Sigma‐Aldrich) solutions were added to *P. marinus* cultures treated with or without 50 μM fluridone to yield the indicated final concentrations. ABA was dissolved in DMSO at 100 mM. Lycopene and β‐carotene were dissolved in tetrahydrofuran (THF) (Wako Pure Chemical Industries) at 2 mM immediately before each experiment. The amount of DMSO and THF added to the cells did not exceed 0.1% (v/v) and 0.5% (v/v), respectively.

### Fluorescence microscopy

Morphological influence of fluridone was observed using cells treated with or without 50 μM fluridone for 3 d. The selected concentration was just above the IC_50_, thereby facilitating the observation of any associated changes. Cells were then heat treated at 26 °C or 60 °C for 30 min, and subsequently incubated at 37 °C for 10 min with the combination of 5 μg/ml Bisbenzimide H 33342 Fluorochrome (Hoechst 33342) and 5 μg/ml propidium iodide (PI). Images were recorded using an Axio Imager 2 microscope (Carl Zeiss, Jena, Germany) and processed with ImageJ software (National Institutes of Health, Rockville, MD).

### ABA extraction

*Perkinsus marinus* cells were collected from a two‐liter culture by centrifugation at 2,000 *g* for 20 min at 4 °C and washed with ATCC medium 1886 without lipid mixture, JLP carbohydrate, or fetal bovine serum. The pellet was weighed and resuspended in 240 ml of ice‐cold 80% methanol containing 500 mg/l citric acid monohydrate (Wako Pure Chemical Industries) and 100 mg/l butylated hydroxytoluene (Wako Pure Chemical Industries) as antioxidants. After stirring overnight at 4 °C in the dark, the suspension was centrifuged at 1,500 *g* for 20 min at 4 °C; the supernatant then was evaporated to dryness and the extract was redissolved in 5 ml of 20% methanol containing 0.1% formic acid (Wako Pure Chemical Industries). The impurities in the solution were removed as described previously (Lewis and Visscher 1982), with minor modifications. Briefly, the Sep‐Pak C18 cartridge (Waters Corp., Milford, MA) was conditioned with 100% methanol and equilibrated with 20% methanol containing 0.1% formic acid, and 5 ml of the extracted solution was loaded onto the cartridge. The cartridge was then washed with 10 ml of 20% and 32% methanol containing 0.1% formic acid, and eluted with 10 ml of 50% methanol. The eluates were evaporated to dryness and the residue was resuspended in 0.5 ml of 50% methanol containing 0.1% formic acid. The suspensions were filtered through a 0.45‐μm Ultrafree‐MC centrifugal filter unit (Merck Millipore, Billerica, MA).

For the parasite grown in the presence of 100 μM ABA, a three‐day culture in a 500‐ml medium was collected and was washed three times with 40 ml per wash of ATCC medium 1886 to thoroughly remove extracellular ABA. The pellet then was extracted and purified as described above. The residue of the dried eluate was resuspended in 0.1 ml of 50% methanol containing 0.1% formic acid, and filtered as above.

### LC‐MS analysis

The filtrate was analyzed using a liquid chromatography mass spectrometry (LC‐MS) system JMS‐T100LP (JEOL, Tokyo, Japan), with an electrospray ionization (ESI) source (negative mode). The separation was carried out on an Inertsil ODS‐3 (3 mm i.d. × 10 cm, 5 μm) (GL Science, Tokyo, Japan). The column was eluted with 50% methanol containing 0.1% formic acid at 0.3 ml/min. The capillary voltage was 2.5 kV and mass spectra were acquired in the range of 50–1,000 *m/z*. Ten microliters of standard solutions of ABA (Sigma‐Aldrich) and of each of the cell samples were analyzed using this system, and the mass chromatograms of *m/z* 263 were plotted for 10 min. To verify whether the cell sample suppresses ionization efficacy, the ABA standard was spiked into the cell sample at a concentration corresponding to the limit of detection prior to analysis.

## Results

### The carotenoid‐ABA biosynthesis inhibitor blocks *Perkinsus marinus* cell growth

**Figure 2:**
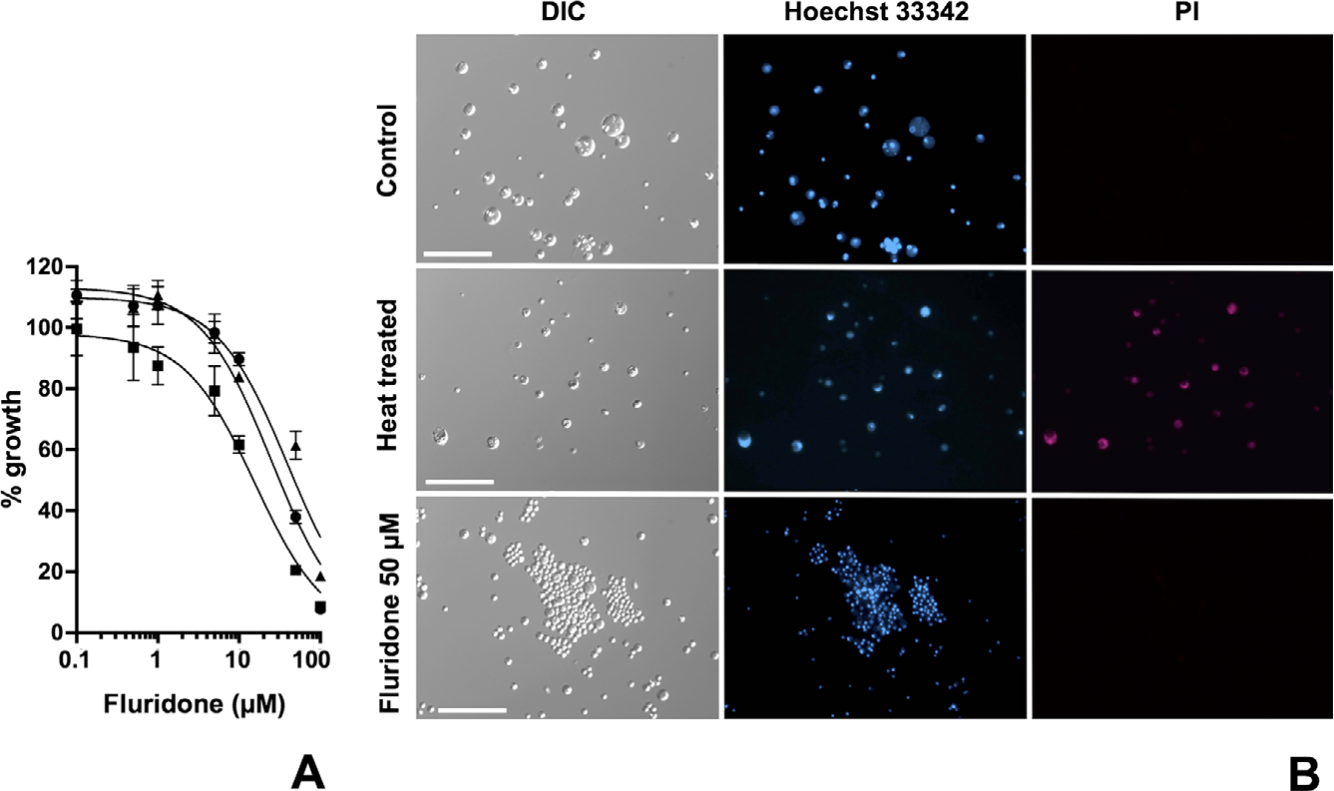
Effect of fluridone on growth of *Perkinsus marinus* in in vitro culture. (**A**) Growth was inhibited by fluridone (0.1–100 μM) in a concentration‐dependent manner (IC_50_ = 24.9 μM, 95% CI = 14.7–42.3 μM). Three independent assays (represented with different lines and markers) were performed in triplicate shown as mean ± standard deviation (SD). (**B**) Nuclear staining with Hoechst 33342 and propidium iodide (PI). Heat‐treated cells (at 60 °C) served as a positive control for PI staining. No PI fluorescence was observed in the cells treated with 50 μM fluridone. Scale bars, 50 μm.

We first confirmed the inhibitory activity of the ABA biosynthesis inhibitor fluridone against *P. marinus* because the parasite strain and growth assay system used in our work differed from that used in the previous study (Shridhar et al. 2013). On the third day of culture, fluridone was found to have inhibited cell growth in a dose‐dependent manner (Fig. 2A), although the growth did not reach the bottom plateau at the highest tested concentration of fluridone. Data for higher concentrations of fluridone could not be collected due to the compound's insolubility at concentrations exceeding 100 μM; hence, to estimate IC_50_ value, we conjectured that growth converges on 0% at higher concentrations of the inhibitor. The mean IC_50_ value of three independent assays was 24.9 μM (95% CI: 14.7–42.3 μM).

A nuclear stain was performed using PI, which is not permeable to live cells, to show whether the treated *P. marinus* cells were dead or alive (Fig. 2B). The cells treated with fluridone were stained after the growth assay, and untreated and heat‐killed cells were used as negative and positive controls, respectively. Although live‐cell‐permeable Hoechst 33342 signals were observed in every cell, PI fluorescence was not observed in the cells treated with 50 μM fluridone (Fig. 2B). Even with longer (for 7 d) or higher concentration (100 μM) treatments, PI fluorescence was not observed (data not shown). This result indicated that fluridone does not alter the membrane integrity of the parasite. Indeed, the parasite resumed growth after washout of the inhibitor, although fluridone treatment caused retardation for the first 2 d postwashout (Fig. S1A). These results indicated that fluridone does not kill the parasite but does arrest growth. It is noteworthy that fluridone induced extraordinary cell clustering compared to the control culture (Fig. 2B), and that the clustering of cells decreased markedly following removal of the inhibitor (Fig. S1B).

### *Perkinsus marinus* is insensitive to ABA and its intermediates

**Figure 3:**
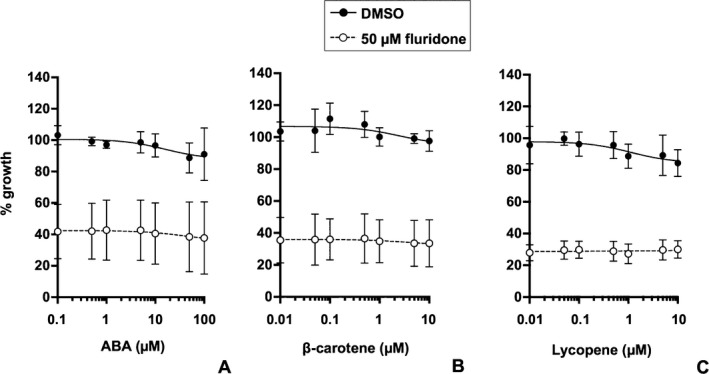
Rescue assay employing treatment with ABA and intermediate carotenoids. ABA and carotenoids were added to the parasite culture treated with DMSO as a control (solid lines and closed circles) or 50 μM fluridone (dashed lines and open circles). (**A**) ABA, 0–100 μM; (**B**) β‐carotene, 0–10 μM; (**C**) lycopene, 0–10 μM. Three independent assays were performed in triplicate, and the data are shown as mean ± SD.

To assess whether the inhibition could be attributed to decreased ABA production, we performed a rescue assay using ABA and its intermediates. Contrary to our expectations, exogenous ABA did not affect parasite growth in the presence of 50 μM fluridone (Fig. 3A). It is possible that ABA intermediates (Fig. 1), but not ABA itself, are important for parasite growth. However, supplementation with β‐carotene (Fig. 3B) and lycopene (Fig. 3C) also did not influence the growth, although β‐carotene was apparently taken up by the cells (Fig. S2).

### Determination of ABA content using mass spectrometry

**Figure 4:**
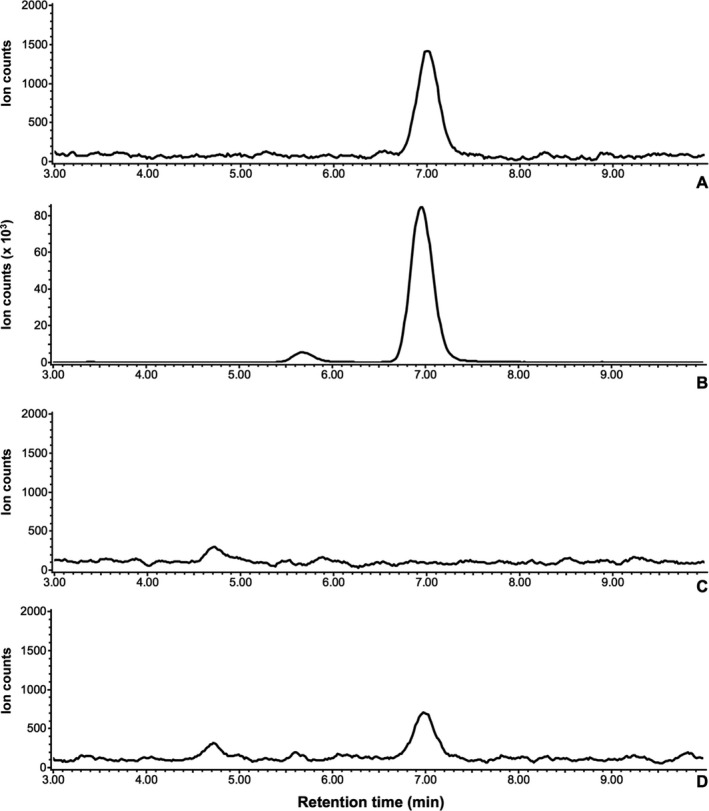
Comparison of mass chromatograms of *m/z* 263 between the standard and the parasite cell sample. (**A**) 0.25 ng of ABA standard. (**B**) The parasite cell sample cultured with 100 μM ABA. (**C**) The parasite cell sample cultured without exogenous ABA. (**D**) 0.25 ng of ABA standard spiked into the parasite cell sample (C).

We analyzed ABA uptake and production in *P. marinus* cells using the LC‐MS system because the growth assays did not demonstrate any significant role for ABA in parasite growth and suggested that little or no ABA had been produced. In standard samples, the *m/z* 263 ion peaks of 0.025 (Fig. 4A), 0.05, 0.1, and 1.0 ng/μl of ABA were detected by the system at 7.0 min, while the ion peak of 0.01 ng/μl was not detected. Regression analysis showed that the *R* ^2^ value was 0.99993 in the range of 0.025–1.0 ng/μl. Thus, we decided that the limit of detection was 0.025 ng/μl.

We first examined the uptake of ABA into the parasite cells to corroborate the result of the rescue assay. From the 500‐ml culture with 100 μM ABA, we obtained 1.74 g fresh weight (f.w.) of cell pellet after washing. An ion peak at *m/z* 263 was detected at 7.0 min from the extract of this pellet (Fig. 4B), a peak that corresponded to that of the ABA standard (Fig. 4A). Notably, the detected concentration was far higher than the upper quantitation limit (1.0 ng/μl). The maximal carry‐over of ABA from medium into the extract was estimated as 0.033 ng/μl; this inference was based on the presumption that the 1.74 g f.w. of cell pellet itself carried 1.74 ml supernatant at most during each wash with 40 ml medium. Thus, the detected ABA appeared to be derived from the parasite cells, showing that the exogenous ABA can be incorporated into the cells and that intracellular ABA is measurable by our experimental procedures.

ABA production in the parasite was then examined. At least 7.8 g f.w. of parasite cells was prepared from a two‐liter culture. The ion peak at *m/z* 263 was not detected at 7.0 min from the extract (Fig. 4C). Since the peak was clearly detected when ABA standard was spiked into the extract at 0.025 ng/μl (Fig. 4D), any ion suppression effect of the cell extract had to be having only a small impact in our experimental setting. The limit of detection in cell extract was still 0.025 ng/μl, despite the observed suppression of ionization.

We also attempted to detect the predicted ABA metabolites to exclude the possibility that ABA was oxidized in the parasite cell. The major oxidative pathway of ABA in Embryophyta is 8′‐hydroxylation by a cytochrome P450 707A (CYP707A); this process forms 8′‐hydroxy ABA, which is then spontaneously isomerized to phaseic acid (PA) and reduced to dihydrophaseic acid (DPA) (Nambara and Marion‐Poll 2005). These metabolites can be extracted with methanol (Tureckova et al. 2009); we therefore searched the *m/z* 279 (for PA) and 281 (for DPA) peaks in the cell extract. These peaks were not detected (data not shown) indicating that the lack of ABA in the extract was not attributable to the oxidation.

## Discussion

ABA is commonly found in plants, algae, and cyanobacteria (Hartung 2010; Nambara and Marion‐Poll 2005), as well as in nonphotosynthetic holoparasitic plants such as *Orobanche hederae* (Ihl et al. 1987) and *Orobanche minor* (Chae et al. 2004). The major function of ABA is induction of dormancy due to arrest of cell activities. Interestingly, the apicomplexan parasite *T. gondii* also produces ABA, but its function is opposite to that in higher plants. In *T. gondii*, ABA facilitates cell growth, and the ABA biosynthetic inhibitor fluridone induces dormancy, that is, cyst formation (Nagamune et al. 2008). In this study, we confirmed that fluridone also inhibits *P. marinus* cell growth (Fig. 2A). The IC_50_ value was comparable to that obtained in a previous study using a different assay system and strain (IC_50_ = 38 μM on day four of culture) (Shridhar et al. 2013). The sensitivity of *P. marinus* to fluridone was of the same order as that in *T. gondii* (IC_50_ = 15 μM) (Nagamune et al. 2008), and was similar to the sensitivity of *P. falciparum* to norflurazon, another ABA biosynthetic inhibitor (IC_50_ = 25 μM) (Tonhosolo et al. 2009). Furthermore, the fluridone‐treated *P. marinus* cells were not killed but instead arrested (Fig. 2B, S1A), with cell clustering observed in the fluridone‐treated culture (Fig. 2B, S1B). Clustering also is observed in cultures that have not been diluted or fed about for a week (Sunila et al. 2001), suggesting that the clustering may reflect a stationary phase response to stresses and perhaps has relationships in underlying mechanism to cyst formation observed in fluridone‐treated *T. gondii* (Nagamune et al. 2008). These results alone may be taken to indicate that *P. marinus* also has an ABA biosynthesis pathway and that ABA plays an important role in parasite growth, as is seen in the apicomplexan parasites.

Contrary to the results of the growth assay, exogenous ABA did not affect the parasite growth, irrespective of fluridone treatment. Even 100 μM ABA or 10 μM of intermediate carotenoids did not rescue fluridone‐inhibited growth (Fig. 3), contrasting starkly with previous results whereby 10 μM ABA and 200 nM lycopene were sufficient to rescue *T. gondii* (Nagamune et al. 2008) and *P. falciparum* (Tonhosolo et al. 2009), respectively. When the *P. marinus* cultures were supplemented with 100 μM ABA, more than 217 pmol/g f.w. ABA was incorporated into the parasite cells (Fig. 4B). Carotenoids also can be incorporated into the parasite cells; given that the medium contains Tween 80 at levels exceeding the critical micelle concentration, carotenoids would be solubilized into micelles and then absorbed into cells by diffusion, as seen in mammalian intestinal cells (Sugawara et al. 2001). Indeed, accumulation of exogenous carotenoids was observed in the parasite cells (Fig. S2). These observations indicated that the growth of parasite cells was not rescued, despite the incorporation of ABA and intermediate carotenoids, suggesting in turn that fluridone's effect on growth was not the result of inhibition of the biosynthesis of these compounds. This interpretation also is supported by the mention of Shridhar et al. (2013), who noted without data that the two candidate genes for phytoene desaturase (PDS), which are required for carotenoid synthesis and are targets of fluridone (Gamble and Mullet 1986), are not expressed in trophozoites under standard culture conditions and do not have the typical bipartite signal for plastid localization. These data imply that there is a novel target of fluridone in the parasite.

Finally, we could not detect ABA or its metabolites in *P. marinus* (Fig. 4). The detection limit of ABA in our experimental setting was 0.025 ng/μl, corresponding to 5.99 pmol/g f.w. in the cell pellet, and we consider that ABA content in the parasite is significantly lower than the value. Such a level would be lower than the ABA concentration in algae and unstressed plant leaves (7–34 and 50 pmol/g f.w., respectively) (Hartung 2010). In combination with the absence of PDS expression mentioned by Shridhar et al. (2013), our data indicate that this organism does not produce a functional amount of ABA and that the growth of this parasite is not governed by the levels of carotenoids or ABA. Thus, these results suggest that ABA does not have a significant role in parasite growth.

In conclusion, we demonstrated for the first time that the physiological significance of ABA is not conserved between *P. marinus* and apicomplexans. Our findings highlight the divergence of a cell cycle regulation system and plastid function among parasites, a distinction that may be associated with the independent loss of photosynthetic ability in these two lineages. To understand the significance of the retention of nonphotosynthetic plastids, a comparison of the metabolites derived from IPP (e.g. ubiquinones necessary for respiratory chain activity, prenylated proteins such as Rab GTPases, and dolichols required for glycosylphosphatidylinositol anchor biosynthesis, etc.) will be required. Given the dearth of functional analyses of organisms that harbor nonphotosynthetic plastids, with the exception of *Plasmodium* spp. and *T. gondii*, it will be important to focus on other organisms to fill this gap in our knowledge.

## Supporting information


**Figure S1.** *Perkinsus marinus* cells after removal of fluridone.
**Figure S2.** Incorporation of exogenous β‐carotene into *P. marinus* cells.
