# Supplementary material for: Investigation into the Physiological Significance of the Phytohormone Abscisic Acid in Perkinsus marinus, an Oyster Parasite Harboring a Nonphotosynthetic Plastid
Source: J Eukaryot Microbiol. 2016 Nov 28;64(4):440–6. doi: 10.1111/jeu.12379 (PMC5573998; doi:10.1111/jeu.12379)
Supplement: Supplementary file 1 — Figure S1. Perkinsus marinus cells after removal of fluridone. Figure S2. Incorporation of exogenous β‐carotene into P. marinus cells. [file JEU-64-440-s001.pdf]

**SUPPORTING INFORMATION**

**Investigation into the Physiological Significance of the Phytohormone Abscisic Acid in *Perkinsus marinus*, an Oyster Parasite Harboring a Non-Photosynthetic Plastid** by Hirokazu Sakamoto, Shigeo Suzuki, Kisaburo Nagamune, Kiyoshi Kita, Motomichi Matsuzaki

## Supplemental Methods

**Figure S1.** *P. marinus* cells after removal of fluridone. After 3 d treatment with fluridone, the parasites were washed and diluted with fresh medium to $OD_{600}$ = 0.1. **(A)** Growth of the parasites after fluridone removal. Growth was monitored at $OD_{600}$, and the error bars indicate SD from three independent assays. Circle, vehicle control; square, 50 µM fluridone; triangle, 100 µM fluridone. **(B)** Cell clustering of the parasites. The parasites before (day 0) and after (day 3) fluridone removal were imaged under a light microscope with differential interference contrast. Scale bars, 50 µm.

**Figure S2.** Incorporation of exogenous β-carotene into *P. marinus* cells. **(A)** Color changes of cells and their cytosolic fraction. The cells cultured with β-carotene displayed an orange color, and the cytosolic fraction (supernatant) also was colored. **(B)** Difference spectrum between the cytosolic fractions prepared from cells cultured with and without β-carotene. Two absorption maxima observed at 450-500 nm, a range typical of β-carotene. This result indicated that β-carotene was incorporated not only into cell membranes but also into the cytosol.

## Supplemental Methods

*Proliferation of fluridone-treated P. marinus after washout of fluridone.*

Fluridone-treated *P. marinus* was observed after fluridone removal to elucidate whether the inhibitor kills the parasite or not. Cell growth was monitored via optical density at 600 nm ($OD_{600}$) using a DU730 spectrophotometer (Beckman Coulter, Franklin, NJ). The parasite cells were first diluted in 10 ml medium to give $OD_{600}$ = 0.1, and then cultured with or without fluridone for 3 d. The parasites were collected by centrifugation at 800 *g* for 5 min at room temperature and washed three times with 1 ml/wash of ATCC medium 1886 to thoroughly remove fluridone. The washed cells were diluted in 15 ml medium to give $OD_{600}$ = 0.1, and then cultured for 5 d. Images were recorded using an Axio Imager 2 microscope (Carl Zeiss, Jena, Germany) and processed using ImageJ software (National Institutes of Health, Rockville, MD).

*Determination of β-carotene uptake be* P. marinus *cells.*

The parasite cells cultured in 250 ml medium with or without 10 µM β-carotene for 3 d were harvested by centrifugation at 2,000 *g* for 20 min at 4 °C. The cell pellet was washed three times with 40 ml/wash of ATCC medium 1886 to thoroughly remove extracellular β-carotene. Cells

resuspended in phosphate-buffered saline were disrupted at a pressure of 180 MPa using a French Press (Ohtake, Tokyo, Japan). The disrupted cells were first centrifuged at 40,000 *g* for 20 min at 4 °C to remove cell debris and major organelles, and the resulting supernatant was ultra-centrifuged at 200,000 *g* for 60 min at 4 °C to obtain the cytosolic fraction. Carotenoids in the cytosolic fraction were detected by measuring the UV spectrum using a U-660 Spectrophotometer (JASCO, Tokyo, Japan).

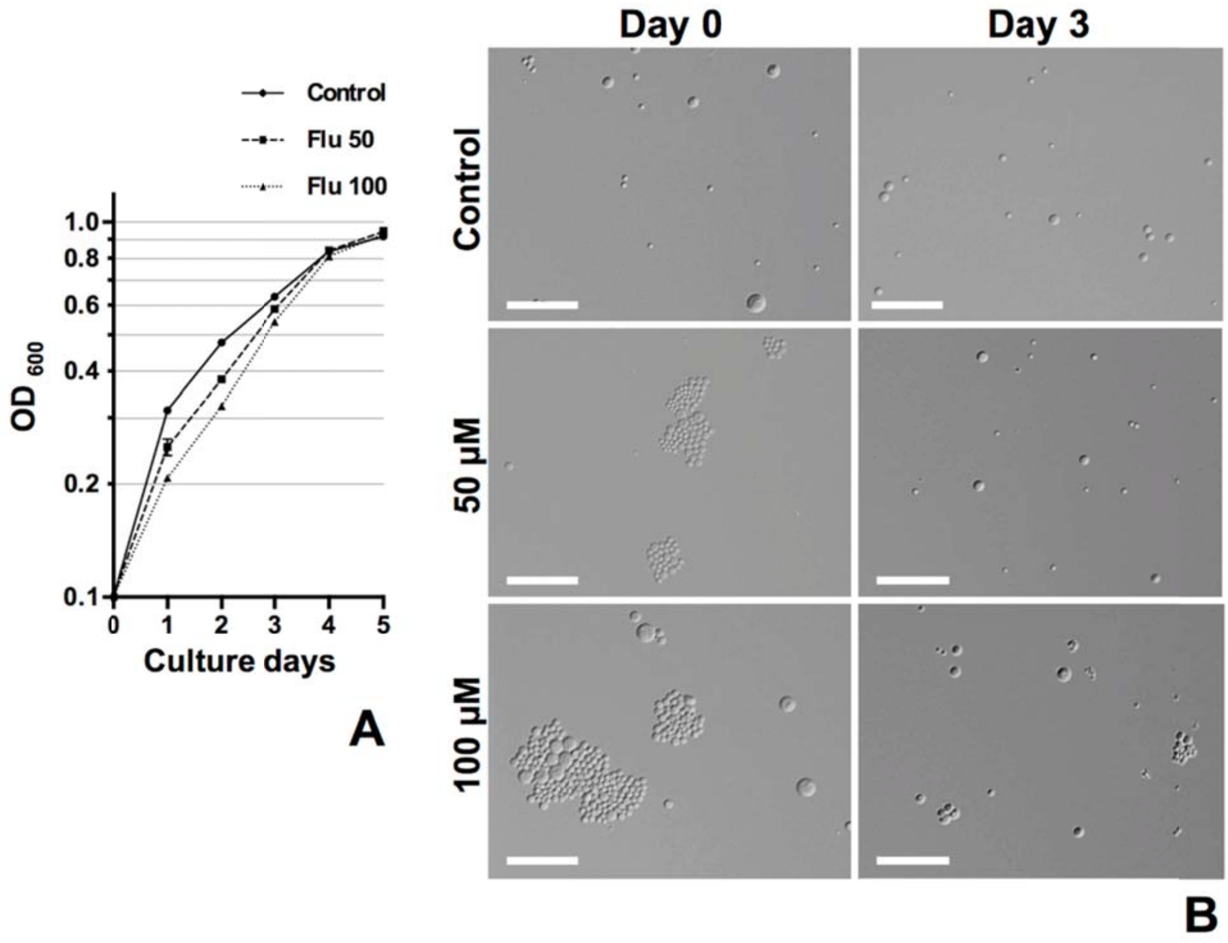

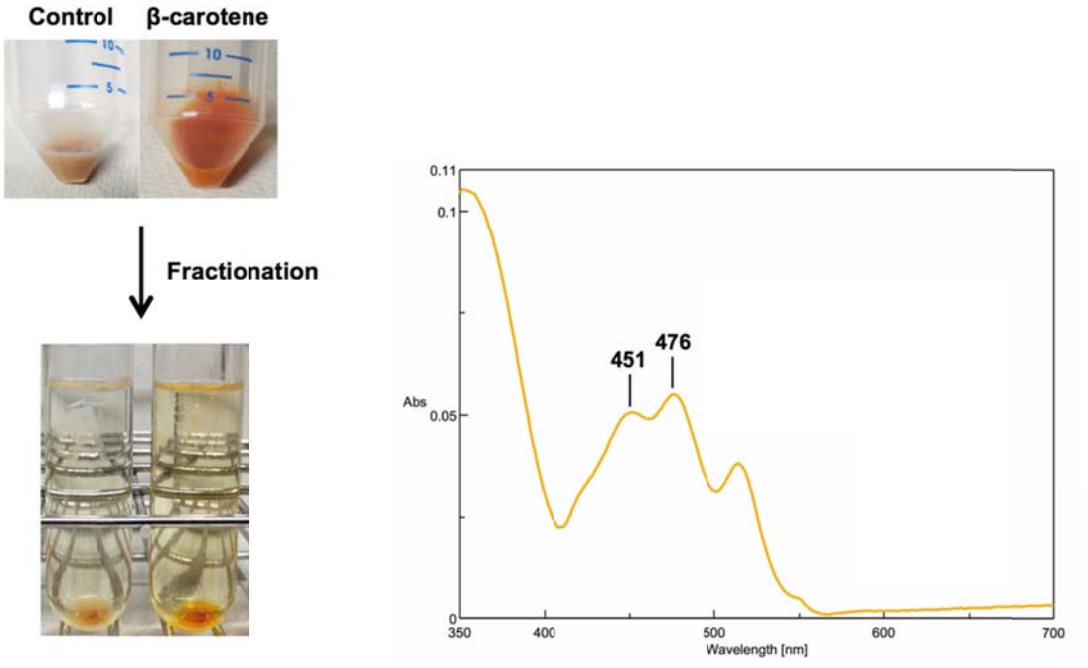
Control
β-carotene
Fractionation
Abs
0.11
0.1
0.05
0
350
400
500
600
700
Wavelength [nm]
451
476

A B
